# Exploring Hückel Molecular Orbital Energies through Variational and Phase Estimation Quantum Algorithms

**DOI:** 10.1021/acs.jpclett.5c03857

**Published:** 2026-02-16

**Authors:** Da Bean Han, Kang-Min Hu, Hyang-Tag Lim, Hyun Woo Kim

**Affiliations:** † Department of Chemistry, 65419 Gwangju Institute of Science and Technology (GIST), Gwangju 61005, Republic of Korea; ‡ Center for Quantum Technology, 58975 Korea Institute of Science and Technology (KIST), Seoul 02792, Republic of Korea; § Division of Quantum Information, KIST School, Korea University of Science and Technology, Seoul 02792, Republic of Korea

## Abstract

Recent advances in quantum technologies have led to the development of several quantum algorithms for computing molecular energetics. However, most existing approaches are limited to determining ground-state energies with relatively few studies addressing multilevel systems. In this work, we explore two quantum algorithms capable of addressing multilevel molecular orbital (MO) energetics: the subspace search variational quantum eigensolver (SSVQE) and iterative quantum phase estimation (IQPE). To benchmark their performance, we employed an exactly solvable Hamiltonian derived from the Hückel method. Both SSVQE and IQPE successfully reproduced the MO energies. We further discussed quantum circuit design and measurement noise using SSVQE. We found out the critical influence of quantum circuit design on computational accuracy. By examining SSVQE under noisy conditions, we could discuss its feasibility for implementation on near-term quantum hardware.

## Graphical abstract

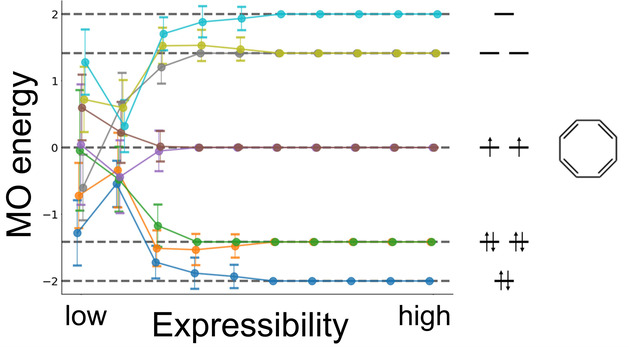

Understanding chemical reactivity through molecular and electronic structure has long been a central aim in chemistry. Achieving this goal relies on quantitative understanding of molecular orbitals (MOs), particularly the frontier molecular orbitals (FMOs) such as highest occupied molecular orbital (HOMO), lowest unoccupied molecular orbital (LUMO), and singly occupied molecular orbital (SOMO). From canonical pericyclic reactions to more complex chemical transformations, FMOs provide powerful and intuitive frameworks for understanding selectivity and kinetics.^1–7^ Excited-state calculations are inherently more complex than ground-state calculations, posing substantial challenges for multilevel analyses in chemistry and materials science. To address these challenges, a variety of advanced electronic-structure methods have been developed and widely applied.^8–10^ Full configuration interaction (FCI) serves as an exact benchmark for electronic-structure calculations, but its exponential scaling with active-space size limits its applicability to small systems. Time-dependent density functional theory (TDDFT) is widely used for larger systems, however it still struggles to deliver accuracy and robustness required for reliable multilevel energy calculations.^11–14^ Quantum computing has emerged as a potential solution to address these limitations.^15,16^

Recent studies suggest that quantum computers could offer practical speed-ups for chemical applications, including molecular property calculations,^15–17^ reaction-path exploration,^18–20^ and drug discovery.^21,22^ In theoretical chemistry, quantum simulations of molecular Hamiltonians are attracting increasing attention, particularly for evaluating both ground and excited-state properties.^23–28^ Quantum phase estimation (QPE) is expected to offer quantum advantage for solving Hamiltonian eigenvalue problems.^29,30^ QPE estimates the eigenphase of a unitary operator *U* = *e* ^–*iHt*^ with high precision. For an eigenstate |ψ_*k*_⟩ of *H* with *H* |ψ_*k*_⟩ = *E*_*k*_ |ψ_*k*_⟩, then *U*(τ) |ψ_*k*_⟩ = *e* ^–*iE*_*k*_τ^ |ψ_*k*_⟩ = *e* ^*i*2*πϕ*_*k*_^ |ψ_*k*_⟩ with $\phi_{k}=$ $-\frac{E_{k}\tau }{2\pi }\left(\mathrm{mod}1\right)$ . In the current noisy intermediate-scale quantum (NISQ) era, the number of controlled-unitary operations required to achieve a given precision, along with resulting circuit depth, makes QPE challenging for realistic chemical systems.^31–34^ To this end, iterative QPE (IQPE) reduces circuit depth by using a single ancilla qubit repeatedly and classical feedback to estimate the phase bits sequentially.^35^ After *m* iterations, IQPE yields ϕ̃_*k*_ with precision $O\left(2^{-m}\right)$ , and $E_{k}=-\frac{2\pi }{\tau }{\mathrm{\phi ̃}}_{k}$ (mod 2π/τ). Choosing τ small enough prevents phase wrapping across the spectrum of *H*. Although recent studies have reported IQPE in quantum chemical calculations for small molecules,^36–38^ the large number of controlled operations remains a major obstacle in the NISQ era. Alternatively, variational quantum algorithms (VQAs) based on the variational principle have become the standard for quantum chemistry due to their relatively shallow circuits and hybrid quantum-classical optimization. Among VQAs, variational quantum eigensolver (VQE) is widely used for ground-state energies,^23,24,26^ and several VQE-based algorithms such as variational quantum deflation,^39^ orthogonally constrained VQE,^40^ ΔADAPT-VQE,^41^ folded spectrum VQE,^42^ and subspace-search VQE (SSVQE)^43^ have been proposed to compute excited states. SSVQE can target multiple low-lying eigenstates within a single optimization by applying a single parametrized unitary to a set of mutually orthogonal input states, which makes the algorithm simpler and removes the need for explicit overlap measurements or orthogonal penalty terms. Furthermore, unlike folded spectrum VQE, it acts directly on the original Hamiltonian rather than on (*H* – *ϵI*)^2^, which reduces the number of Pauli terms and the associated measurement overhead. However, the measurement overhead still grows with the number of target states, and the ansatz should be sufficiently expressive to capture these states. Therefore, the quantum circuit must be designed efficiently to remain practical on NISQ hardware.

Recent studies have already explored quantum computation of HMO energies. Yoshida et al. demonstrated hardware-level HMO energy evaluation using a direct mapping type implementation of the Hückel system,^44^ and Ato et al. extended hardware demonstrations to linear polyenes C_*N*_H_*N*+2_ and reported experimental results for *N* ≤ 6.^45^ Additionally, Singh et al. combined compact encoding with variational approach to reduce qubit requirement.^46^ In this work, we used the Hückel molecular orbital (HMO) model, which provides exact reference solutions and affords straightforward control over the system size, to systematically analyze quantum algorithms and explore potential improvements. We employed weighted SSVQE to compute full HMO energies spectrum simultaneously for linear and cyclic polyenes containing 2^*n*^ carbon atoms, encoded in *n* qubits (2 ≤ *n* ≤ 5). The HMO model adopts a semiempirical π-electron Hamiltonian, provides a compact, analytically solvable reference model, and supports low-qubit, shallow-circuit encodings. HMO theory approximates MOs as linear combinations of atomic orbitals (AOs). It is particularly useful for planar conjugated molecules with σ – π separation, enabling prediction of π-electron MOs and associated energies in π-delocalized systems. We provide a benchmark of SSVQE and IQPE for the Hückel Hamiltonian with compact encoding, enabling evaluation of the full 2^*n*^-level molecular orbital spectrum. We analyze how ansatz depth and parameter initialization influence convergence, and show that noise gives rise to an optimal circuit depth due to a trade-off between expressibility and noise.

In the HMO theory, the *k*-th MO is written as

1 $$\left|\psi_{k}\right\rangle =\overset{N}{\underset{i=1}{\sum }}c_{ik}\left|\phi_{i}\right\rangle$$

where {|ϕ_*i*_⟩} denotes the carbon 2p_*z*_ AOs. Substituting eq 1 into the time-independent Schrödinger equation, *H*|ψ_*k*_⟩ = *E*_*k*_|ψ_*k*_⟩, yields the secular equations:

2 det(H−ES)=0

where *H*_*ij*_ = ⟨ϕ_*i*_|*H*|ϕ_*j*_⟩ and *S*_*ij*_ = ⟨ϕ_*i*_|ϕ_*j*_⟩. With additional empirical assumptions

$$S_{ij}=\delta_{ij},H_{ii}=\alpha ,,H_{ij}=\{\begin{array}{cc} \beta , & \mathrm{if},i,\mathrm{and},j,\mathrm{are},\mathrm{directly},\mathrm{bonded}\\ 0, & \mathrm{otherwise} \end{array}$$

eq 2 reduces to the following secular determinants for linear and cyclic polyenes, respectively.

3 $$\left|\begin{array}{ccccc} \alpha -E & \beta & 0 & \cdots & 0\\ \beta & \alpha -E & \beta & \ddots & \vdots \\ 0 & \beta & \ddots & \ddots & 0\\ \vdots & \ddots & \ddots & \alpha -E & \beta \\ 0 & \cdots & 0 & \beta & \alpha -E \end{array}\right|=0$$

4 $$\left|\begin{array}{ccccc} \alpha -E & \beta & 0 & \cdots & \beta \\ \beta & \alpha -E & \beta & \ddots & \vdots \\ 0 & \beta & \ddots & \ddots & 0\\ \vdots & \ddots & \ddots & \alpha -E & \beta \\ \beta & \cdots & 0 & \beta & \alpha -E \end{array}\right|=0$$

The resulting HMO eigenvalues for an *N*-site linear polyene are given by

5 $$E_{k}=\alpha +2\beta ,\cos\frac{k\pi }{N+1},k=1,2,...,N$$

and for an *N*-site cyclic polyene,

6 $$E_{k}=\alpha +2\beta ,\cos\frac{2k\pi }{N}$$

where $k=0,1,...,\left\lceil \frac{N}{2}\right\rceil$ and for $k=1,...,\left\lceil \frac{N}{2}\right\rceil -1$ , the corresponding energy levels *E*_*k*_ are doubly degenerate. HMO energies depend explicitly on α and *β. α* sets the reference energy and |β| controls the strength of the interaction between adjacent orbitals. For simplicity, the Hückel Hamiltonian for both linear and cyclic polyenes was constructed with parameters α = 0, and β = 1 in this study. For polyenes with 2^*n*^ carbons, we mapped the 2^*n*^ atomic basis states {|*i*⟩}_*i* = 0_^2^*n*^–1^ to the *n*-qubit computational basis. In this basis, the Hückel Hamiltonian is

7 $$H=\alpha I+\beta \underset{\left\langle i,j\right\rangle }{\sum }\left(\left|i\right\rangle \langle j|+\left|j\right\rangle \langle i|\right)$$

where ⟨*i*, *j*⟩ denotes bonded atom pairs. Using Pauli basis *P*_*n*_ ≡ {*I*, *X*, *Y*, *Z*}^⊗*n*^ = {*P*_*k*_}_*k* = 1_^4^*n*^^, *H* can be expanded as

8 $$H=\overset{4^{n}}{\underset{k=1}{\sum }}c_{k}P_{k},c_{k}=\frac{1}{2^{n}}\mathrm{Tr}\left(HP_{k}\right)$$

For Hückel Hamiltonian in eq 7, the number of nonzero terms increases linearly with the system size *N*.^46^

To obtain HMO energies from quantum devices, we can employ various quantum algorithms. A representative example is the VQE, a hybrid quantum-classical method well suited to the NISQ era.^23,24,26^ Given a Hamiltonian written as a weighted sum of Pauli operators, *H* = ∑_*i*_ *c*_*i*_ *P*_*i*_ with real coefficients *c*_*i*_, a parametrized circuit *U*(θ) is used to prepare a trial state |Ψ⟩ = *U*(θ)|0⟩^⊗*n*^. For a given set of parameters θ, the ansatz state |Ψ­(θ)⟩ is prepared on the quantum device, and the expectation values ⟨Ψ­(θ)|*P*_*i*_|Ψ­(θ)⟩ are measured. The energy *E*(θ) = ∑_*i*_ *c*_*i*_⟨Ψ­(θ)|*P*_*i*_|Ψ­(θ)⟩ is evaluated on a classical computer and θ is updated to decrease *E*(θ). By iteratively updating θ until convergence, VQE yields a parameter set θ* that provides a variational approximation to the ground-state energy and corresponding ground state of the given Hamiltonian. The optimization process of VQE can be expressed as

9 $$\theta^{*}=\underset{\theta }{\arg\min}\underset{i}{\sum }c_{i}\left\langle \Psi \right|U^{†}\left(\theta \right)P_{i}U\left(\theta \right)\left|\Psi \right\rangle$$

Although VQE has been successful for many applications,^24^ estimating excited-state energies remains challenging, as it was originally designed for the electronic ground state. To overcome this, various VQE-based excited-state calculation methods have been proposed.^39,40^ These methods enforce orthogonality between states by running separate optimizations and performing additional measurements. In contrast, SSVQE exploits the fact that unitary operations preserve inner products, enabling simultaneous optimization of multiple states within a single run. Applying a single parametrized circuit *U*(θ) to an orthonormal set of input states {|Ψ_*j*_} yields an orthonormal set of outputs {*U*(θ)|Ψ_*j*_}.^43^ In this study, strongly entangling (SE) layers^47^ containing CNOT gates along with parametrized rotation gates were used as *U*(θ) (Figure 1(a,b)) and circuit expressibility is tuned by the number of SE layers. We can minimize a weighted sum of energies,

10 $$L\left(\theta \right)=\overset{k-1}{\underset{j=0}{\sum }}w_{j}\left\langle \psi_{j}\right|U^{†}\left(\theta \right)\mathrm{HU}\left(\theta \right)\left|\psi_{j}\right\rangle$$

where {*w*_*j*_} is a set of decreasing positive weights *w*_0_ > *w*_1_ >··· > *w*_*k*–1_. If the ansatz is sufficiently expressive, multiple eigenstates can be obtained simultaneously. However, higher expressibility can induce barren plateaus, where the gradient of the cost-function vanishes.^48^ Because SSVQE is iterative and targets multiple eigenstates, repeated circuit evaluations and measurements can constitute a major overhead on NISQ hardware. Both noise-free and noisy scenarios were considered to estimate the accuracy achievable on quantum hardware. All calculations were simulated using the PennyLane library.^49^ In addition, to compare with algorithms applicable in the fault-tolerant quantum computing (FTQC) era, we also used IQPE and compared its results with those from SSVQE.

**Figure 1:**
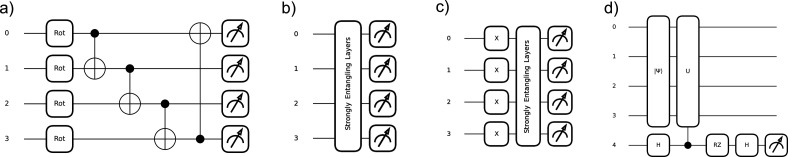
Quantum circuits used in this work. a) Strongly entangling (SE) layer acting on 4-qubit circuit, b) VQE ansatz with input state |0⟩, c) VQE ansatz with input state |15⟩, and d) IQPE circuit.

In hybrid quantum–classical approaches using parametrized circuits, such as VQE and SSVQE, choice of initial parameters can strongly influence the performance of classical optimizers. To evaluate this effect, we performed ten independent runs with distinct random seeds, using the Adam optimizer^50^ for 1000 iterations at a learning rate of 0.01, and reported the mean and standard deviation. We first applied SSVQE to linear and cyclic polyenes to compute their HMO energies. For clarity, linear polyenes are denoted as l-C_*N*_ and cyclic polyenes as c-C_*N*_ where *N* ∈ {2^*n*^|*n* = 1, 2, 3, 4, 5}. For each system, we targeted all 2^*n*^ HMO energies and compared them with the reference values given by eq 5, 6. The accuracy of computed energies was quantified using the root-mean-square error (RMSE), given by

11 $$\mathrm{RMSE}=\sqrt{\frac{1}{N}\overset{2^{n}}{\underset{k=1}{\sum }}{\left(E_{k}^{\mathrm{SSVQE}}-E_{k}^{\mathrm{ref}}\right)}^{2}}$$

where *E*_*k*_^SSVQE^ is the energy calculated using SSVQE and *E*_*k*_^ref^ is the exact solution from eq 5, 6, where *k* indicates the level of HMO energy. For smaller molecules, shallow circuits with a few qubits exhibit limited expressibility, producing a simple optimization landscape where independent runs consistently converge to the same solution, resulting in minimal standard deviations for l-C_2_, l-C_4_, and c-C_4_. However, using too few parameters can limit the achievable accuracy, even when the optimization appeared to have converged globally, as observed for l-C_4_ in Figure 2(a). Increasing the number of SE layers improved the expressibility and accuracy of SSVQE. For larger molecules, both the number of qubits and the states of interest increase, leading to a more complex optimization landscape. With too few SE layers, solutions become more dependent on the initial parameters and are occasionally trapped in local minima. This trend is consistent with the observed increase in standard deviations for l-C_8_, c-C_8_, l-C_16_, and c-C_16_ at small circuit depth. Increasing the number of SE layers enhanced the representational capacity of the circuit, reduced standard deviations, and improved the performance of SSVQE, achieving near-zero RMSE. When the size of polyene increases to C_32_, RMSE does not converge within the range *L* ≤ 10, and requires additional layers to achieve convergence. These findings indicate that circuit capacity is a key factor in the performance of SSVQE-based multilevel energy calculations. In particular, l-C_16_ and c-C_16_ required greater circuit expressibility than smaller polyenes to accurately calculate HMO energies, as shown in Figure 2(c). With more than 20 SE layers, SSVQE achieved near-exact agreement for all 16 HMO energies of l-C_16_ and c-C_16_. To examine the role of initial conditions, we reported additional results in Figure S1 in which the SE layers are replaced with less expressive alternatives, including basic entangler layers and random layers^49^ as shown in Figure S1.

**Figure 2:**
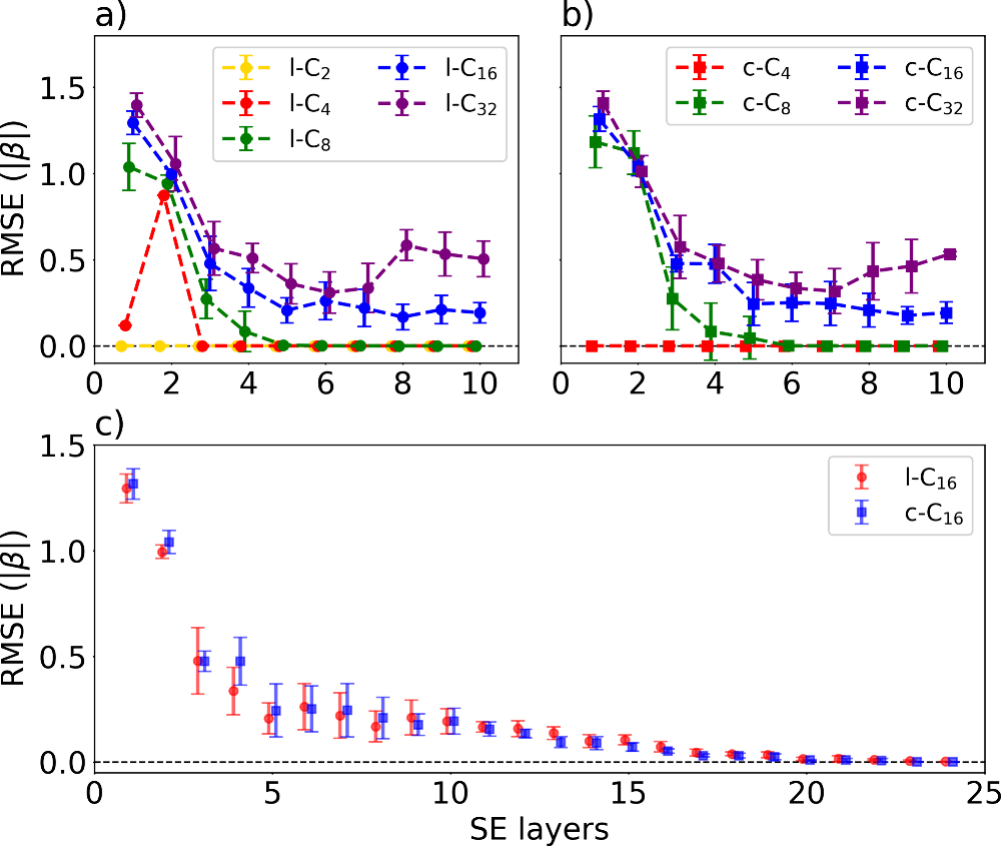
Performance of SSVQE as a function of the number of SE layers for a) linear polyenes, b) cyclic polyenes, and c) l-C_16_/c-C_16_. The error bars show the RMSE in units of |β| as a function of the number of SE layers. The dashed horizontal line indicates zero error (RMSE = 0).

Next, we computed HMO energies using the IQPE algorithm (Figure 3). First, with *m* = 4 phase bits, we evaluated all nine C_*n*_ systems (Figure 3(a)). In all cases, IQPE consistently showed lower accuracy than SSVQE under conditions with a sufficient number of layers. In IQPE, energy resolution is determined by the phase-bit count *m* as $\delta E\approx \frac{2\pi }{\tau }2^{-m}$ so with four phase bits, spacing remains too coarse for the increasingly dense spectra of larger C_*n*_, leading to larger total errors in multilevel energy calculations (Figure 3(a)). To improve the resolution, one should increase *m* and set τ to avoid phase wrapping while retaining fine energy spacing. We therefore increased *m* from *m* = 4 to *m* = 8 and executed C_16_ HMO calculation (Figure 3(b)). Previous work on benzene derivatives suggested that about 12 or more phase bits are required to reach chemical accuracy,^38^ and additional bits should further reduce error. However, the exponentially increasing number of controlled-*U* operations is challenging on present NISQ devices, and standard QPE would additionally require *m*-qubit phase register and an inverse QFT, further increasing depth. Consequently, practical applications with QPE-based methods remain challenging. Qudit-based phase-estimation schemes could, in principle, reduce register width and QFT depth, potentially lowering inverse-QFT costs.^26,51^ However, their realized advantage would depend on device-level limitations. In the present qudit platform, multilevel decoherence and high-fidelity controlled operations are often more challenging. We report one- and two-qubit gate counts and circuit depth for SSVQE and IQPE in Table 1. The comparison highlights the rapid growth of IQPE resource requirements with increasing phase-bit precision. IQPE is also sensitive to the overlap between the input and target states, as low overlap reduces the success probability of accurate phase estimation. Since the Hückel Hamiltonian is exactly solvable, its eigenvectors are known analytically. Thus, we report complementary reduced-overlap tests in Figure S2. The overlap between the initial state and the target eigenstate can be enhanced through chemically motivated state preparation, as demonstrated in.^38^ For these reasons, in the remainder of this letter, we focus on SSVQE as the approach is more readily implementable on NISQ hardware.

**Figure 3:**
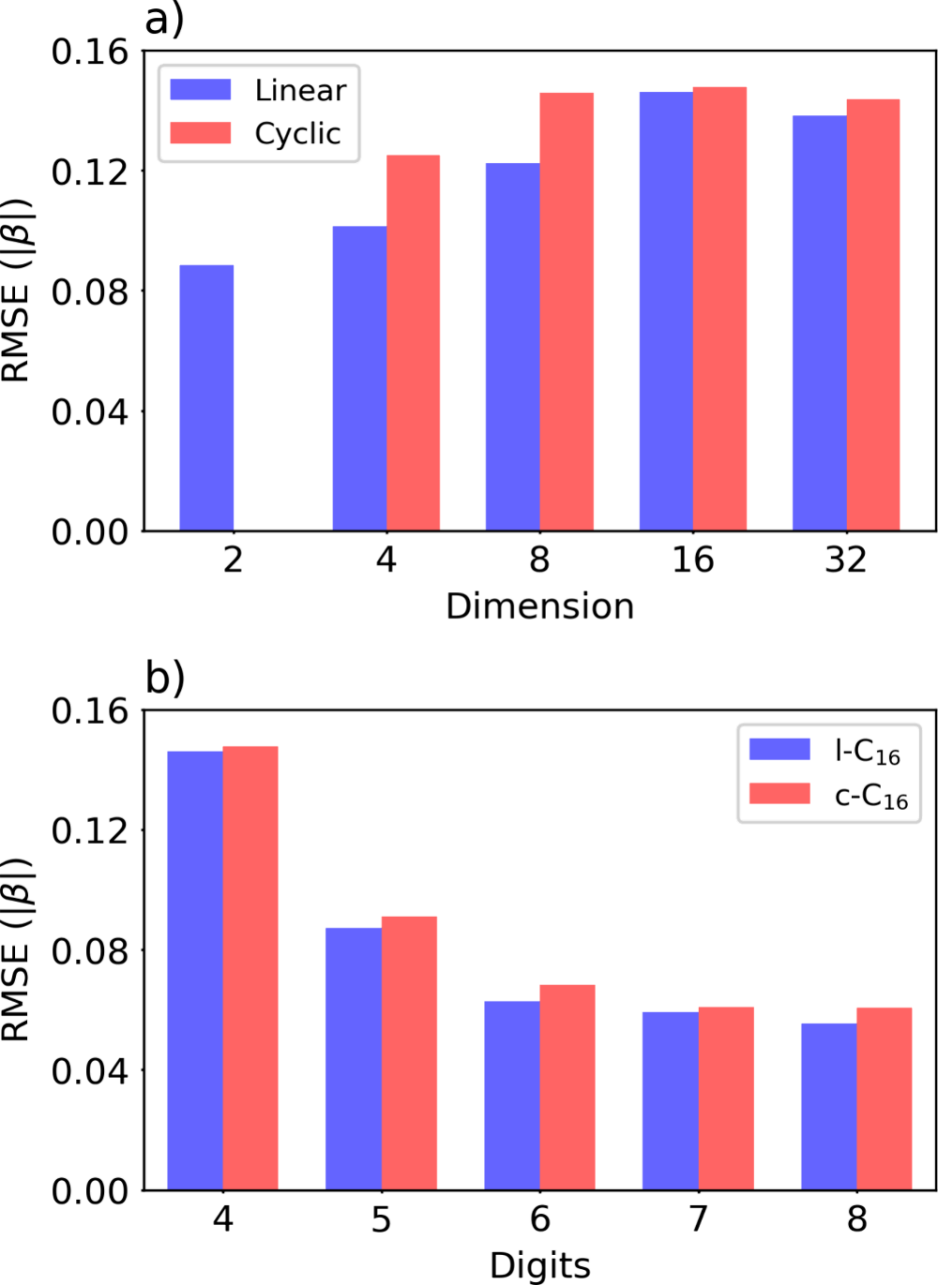
Comparison of IQPE performance a) with four phase bits in all nine polyenes, and b) l-C_16_/c-C_16_ from four to eight phase bits.

**Table 1:** One- and Two-Qubit Gate Counts (*N*_1*q*_ and *N*_2*q*_) and Circuit Depth for SSVQE and IQPE for l-C_8_ and l-C_16_ ^a^

Algorithm	Molecule (Qubits)	Setting	*N*_1*q*_	*N*_2*q*_	Depth
SSVQE	l-C_8_ (3)	*L* = 1	9	3	6
		*L* = 3	27	9	18
		*L* = 5	45	15	30
	l-C_16_ (4)	*L* = 1	12	4	7
		*L* = 3	36	12	19
		*L* = 5	60	20	30
IQPE	l-C_8_ (4)	*m* = 4	23501	10926	22419
		*m* = 5	48536	22574	46301
		*m* = 6	98596	45870	94056
	l-C_16_ (5)	*m* = 4	79189	36854	73194
		*m* = 5	163616	76150	151220
		*m* = 6	332460	154742	307263

a SSVQE uses *L* SE layers, and IQPE uses *m* phase bits. Detailed information is given in Supporting Information.

To gain deeper insight into the effect of circuit design on multilevel MO energy calculations, we further examined l-C_8_ and c-C_8_. Figure 4 shows that, across all HMO levels, accuracy improved as the circuit’s representation capacity increased. Limited expressibility at shallow circuit depth led to reduced accuracy and large dispersion across ten different initializations, whereas with sufficient depth, typically six layers or more, the predicted HMO energies for all eight levels matched the exact HMO energies within an absolute error of 10^–5^|β|. This sensitivity to initialization increases practical cost by necessitating additional optimizer iterations or multistart runs to achieve stable results. For c-C_8_, 2-fold degeneracies were also correctly reproduced as shown in Figure 4(b). Accurate prediction of all HMO levels suggests that SSVQE can aid photochemical reaction analysis and HOMO/LUMO energy calculations for large molecules and materials. The consistent trend in Figure 2 and Figure 4 indicates that increasing the expressibility of the ansatz improves the accuracy of SSVQE. In future applications, the expressibility of the ansatz should be calibrated to the complexity of the target system to accurately calculate multistate energies.

**Figure 4:**
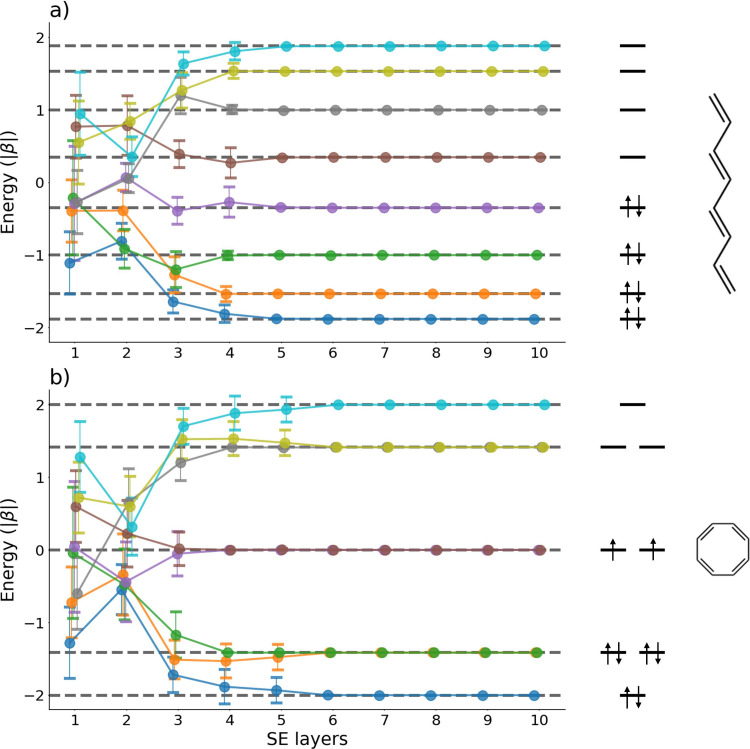
HMO energies of l-C_8_ (a) and c-C_8_ (b) as a function of the number of SE layers. Error bars show calculated HMO energies in units of |β|, and the dashed line indicates the exact HMO energies.

On NISQ hardware, quantum circuits are inherently operated under noisy conditions. Consequently, the resulting gate operations have nonzero error probabilities. Implementing multiqubit gates with high fidelity is more challenging than implementing single-qubit gates, and they often represent a primary performance bottleneck. The SE layer used in this work consists of rotation gates followed by CNOT gates that generate entanglement between qubits. When SE layers are stacked to achieve sufficient expressibility, the number of CNOT operations increases, and implementing them accurately remains challenging. The results in Figures 2 and 4 were obtained with ideal, noise-free circuits. Increasing circuit depth provided higher expressibility and accurate HMO energies under ideal condition. However, on real hardware, device noise and finite coherence times set an upper limit on accuracy, and deeper circuits are further affected by error accumulation, leading to depth-dependent performance degradation. To evaluate the performance of SSVQE under noisy conditions, we computed HMO energies of l-C_8_ using SSVQE in a noise model, and the results are shown in Figure 5. We restricted noise to CNOT gates and, upon each execution, applied an independent single-qubit error channel to the control and target qubits with probability *p*. The noise channel was applied immediately after each CNOT gate and modeled as a single-qubit depolarizing channel. In this model, a depolarization probability *p* ∈ [0, 1] is distributed equally among the Pauli operations, so that each is applied to the qubits with probability *p*/3 while the identity is applied with probability 1 – *p*.^49^ In this case, the fidelity of the CNOT gate is given by

12 $$F_{\mathrm{CNOT}}=\frac{4{\left(1-p\right)}^{2}+1}{5}$$

^52^ We evaluated eight noise levels for the CNOT gate, with corresponding F_CNOT_ ranging from 89.9993% to 99.9992%. Among them, five noise models shown in Figure 5(a) are comparable to or slightly lower than the F_CNOT_ values reported in state-of-the-art experiments.^53–56^ As shown in previous results, in the ideal case RMSE decreased as SE layers are stacked without upper limit. In realistic hardware, however, error accumulation competes with improvement in expressibility. To make this trade-off more apparent, the optimal number of SE layers and corresponding RMSE are shown for each of the eight F_CNOT_ values in Figure 5(b). As the gate fidelity improved, the optimal depth increased from three to eight layers and corresponding RMSE decreased. Additional results including single-qubit and CNOT gate errors show similar trends (Figure S3). The SSVQE was also executed on the IBM quantum computer, *ibm marrakesh*, and the initial stage of the training process was compared with those from ideal and noisy simulations (Figure S4).^57^ Overall, these results highlight a clear trade-off between expressibility and noise that can only be relaxed by higher-fidelity gates and longer coherence times. For the Hückel Hamiltonian, near-term experiments have focused on small polyenes,^45^ with the potential to scale up as two-qubit fidelity and coherence times improve. Since SSVQE requires sufficient circuit depth to achieve accurate results, improved hardware capability is essential. While we enhanced expressibility by stacking SE layers, SE layers can be replaced by alternative and system-optimized parametrized circuits, including physics-guided,^58^ dynamic or adaptive circuits,^59,60^ iterative optimization,^61^ and LLM-guided designs^62^ that improve expressibility at shallower circuit depth.

**Figure 5:**
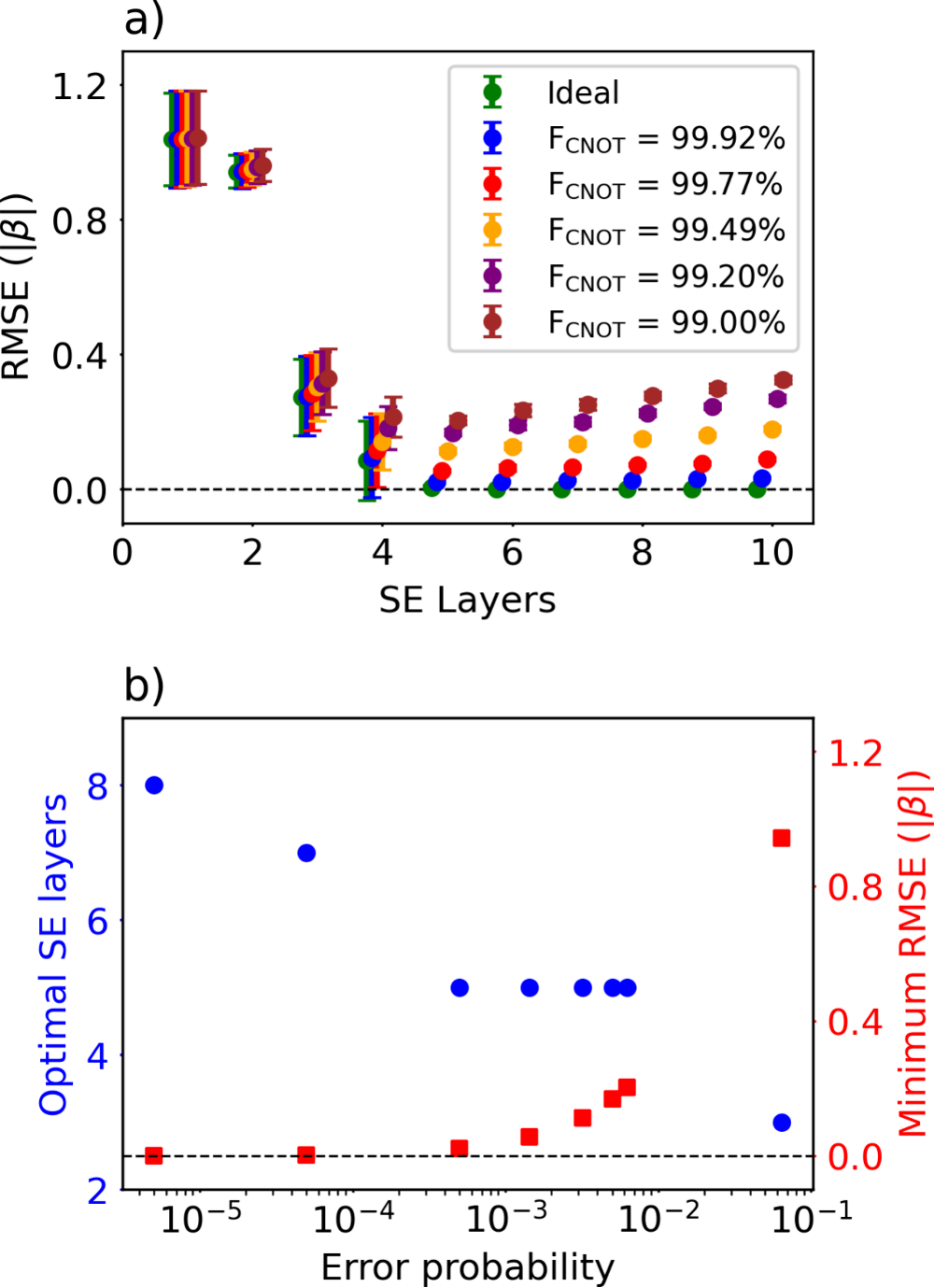
SSVQE results for l-C_8_ obtained in a noisy environment. a) RMSE in unit of |β| as a function of the number of SE layers. Error bars show the mean and standard deviation over ten runs. Results for five noisy CNOT fidelities (F_CNOT_) and ideal (noiseless) result are compared. b) Optimal number of SE layers and the corresponding minimum RMSE for different F_CNOT_. From right to left, F_CNOT_ = 90.0%, 99.0%, 99.2%, 99.5%, 99.8%, 99.9%, 99.99%, and 99.999%.

In summary, we benchmarked two practical quantum algorithms for multilevel MO energy calculations on *n*-qubit representations of linear and cyclic polyenes, using the exactly solvable Hückel model for validation. Although Hückel model does not include electron correlation, it retains key ingredients of quantum chemistry workflow such as Fermionic representation, qubit encoding, and depth-sensitive circuit resources. This benchmark therefore serves as a controlled stepping stone toward correlated Hamiltonians, where classical methods scale exponentially in system size and quantum algorithms may offer a route to improved scaling. SSVQE reproduced MO energy levels with high accuracy when the ansatz was sufficiently expressive. For l-C_8_/c-C_8_ absolute errors were reduced to ≈ 10^–5^ |β| at ≥ 6 SE layers, and for l-C_16_/c-C_16_ near-zero RMSE was achieved with ≥ 20 layers. IQPE could achieve comparable accuracy with sufficient phase bit *m*, however IQPE was impractical on NISQ hardware due to the large number of controlled-unitary operations. By simulating the effect of depolarizing noise, we revealed a clear trade-off between circuit expressibility and noise. While deeper circuits improve performance in the ideal noiseless condition, they become counterproductive on real hardware once multiqubit errors dominate, indicating that the optimal circuit depth is constrained by device fidelity and coherence times. This study provides practical guidance for near-term experiments: (1) SSVQE can serve as a NISQ-practical starting point for multilevel energy calculations when the ansatz expressibility is well matched to system complexity. (2) Circuit depth should be chosen in accordance with error budgets. A systematic exploration of the ansatz families and training protocols may help to achieve the target accuracy at a shallower effective depth. In addition, error-mitigated approach can also be helpful, as demonstrated in error-mitigated quantum simulations of spin-chain dynamics.^63^ (3) IQPE is regarded as a promising FTQC-regime tool where error correction supports long coherent evolutions and high-fidelity controlled operations. Extending this framework to more realistic Hamiltonians, together with advances in simulation methods and quantum hardware, is expected to enable quantitative multistate energy calculations beyond Hückel models in the future. Accurate multistate energy calculations for realistic Hamiltonians could directly support practical applications in which multiple energy levels play a critical role, such as the design of new materials subjected to electric or photonic perturbations.

## Supplementary Material



## Notes

Gate counts and circuit depths for SSVQE and IQPE, Performance comparison of SSVQE across different ansatz types and circuit depths, IQPE performance for varying initial state overlaps, SSVQE results in both noisy and ideal environments (PDF)
