# Supplementary material for: Exploring Hückel Molecular Orbital Energies through Variational and Phase Estimation Quantum Algorithms
Source: J Phys Chem Lett. 2026 Feb 16;17(8):2205–12. doi: 10.1021/acs.jpclett.5c03857 (PMC12951572; doi:10.1021/acs.jpclett.5c03857)
Supplement: Supplementary file 1 [file jz5c03857_si_001.pdf]

Supporting Information: Exploring Hückel Molecular Orbital Energies Through Variational and Phase Estimation Quantum Algorithms

Da Bean Han[1], Kang-Min Hu[2,3], Hyang-Tag Lim[2,3], and Hyun Woo Kim[1,2]

[1]Department of Chemistry, Gwangju Institute of Science and Technology (GIST), Gwangju 61005, Republic of Korea
[2]Center for Quantum Technology, Korea Institute of Science and Technology (KIST), Seoul 02792, Republic of Korea
[3]Division of Quantum Information, KIST School, Korea University of Science and Technology, Seoul 02792, Republic of Korea

*Email: hyangtag.lim@kist.re.kr, hwk@gist.ac.kr

Table S1: One- and two-qubit gate counts (N_{1q} and N_{2q}) and circuit depth for SSVQE and IQPE for l-C_8 and l-C_{16}. SSVQE uses L SE layers, and IQPE uses m phase bits.

Algorithm	Molecule (Qubits)	Setting	N_{1q}	N_{2q}	Depth
SSVQE	l-C_8 (3)	$L = 1$	9	3	6
		$L = 2$	18	6	12
		$L = 3$	27	9	18
		$L = 4$	36	12	24
		$L = 5$	45	15	30
		$L = 6$	54	18	36
		$L = 7$	63	21	42
		$L = 8$	72	24	48
		$L = 9$	81	27	54
		$L = 10$	90	30	60
	l-C_{16} (4)	$L = 1$	12	4	7
		$L = 2$	24	8	12
		$L = 3$	36	12	19
		$L = 4$	48	16	25
		$L = 5$	60	20	30
		$L = 6$	72	24	37
		$L = 7$	84	28	43
		$L = 8$	96	32	48
		$L = 9$	108	36	55
		$L = 10$	120	40	61
IQPE	l-C_8 (4)	$m = 4$	23501	10926	22419
		$m = 5$	48536	22574	46301
		$m = 6$	98596	45870	94056
		$m = 7$	198705	92462	189556
		$m = 8$	398911	185646	380545
	l-C_{16} (5)	$m = 4$	79189	36854	73194
		$m = 5$	163616	76150	151220
		$m = 6$	332460	154742	307263
		$m = 7$	670137	311926	619339
		$m = 8$	332460	626294	1243480

The iterative quantum phase estimation (IQPE) resource estimates are reported only for the lowest-energy state, while subspace search variational quantum eigensolver (SSVQE) resource estimates reports for the full energy spectrum.

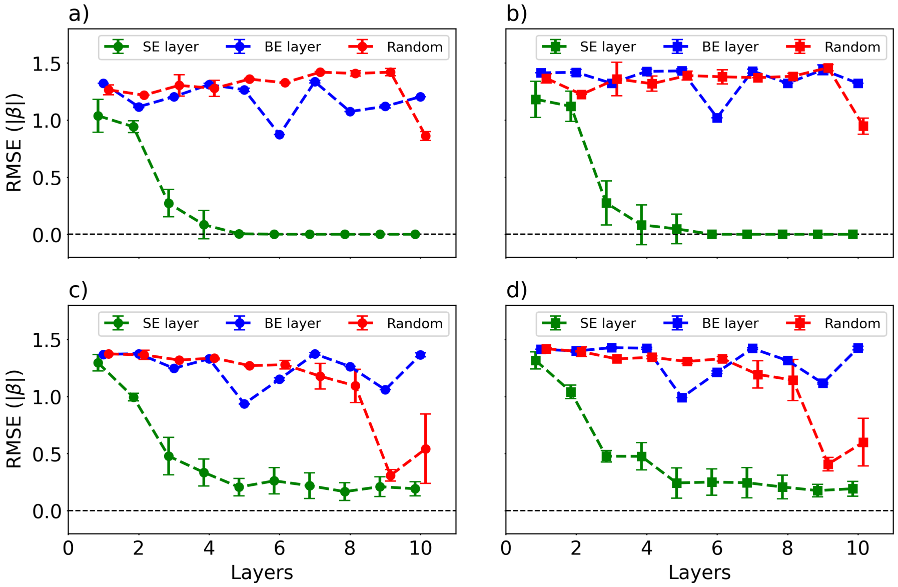

Figure S1: Comparison of SSVQE performance across different ansatz types and circuit depths. Root mean square errors (RMSEs) in unit of $|\beta|$ as a function of the number of layers for (a) l-C_8, (b) c-C_8, (c) l-C_{16}, and (d) c-C_{16} are reported. Three ansatz types are compared: strongly entangling layers (SE layer, green), basic entangler layers (BE layer, blue), and random layer (Random, red) from Pennylane software package.[1] Error bars represent the standard deviation across 10 independent runs with different random seeds (2021-2030). The learning rate and number of iterations are fixed at 0.01 and 1000, respectively.

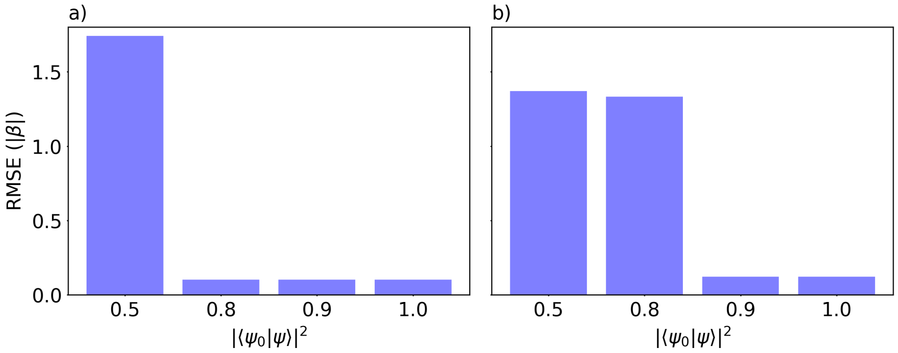

Figure S2: Comparison of IQPE performance a) l-C_4 and b) l-C_8 with four phase bits as a function of overlap between initial state ($|\psi\rangle$) and target eigenstate ($|\psi\rangle$).

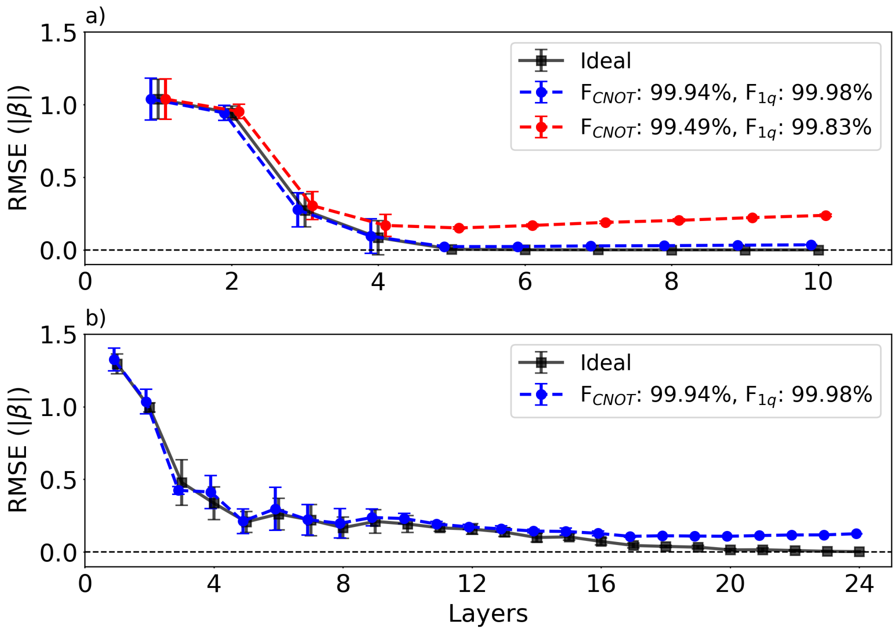

Figure S3: SSVQE results for a) l-C_8 and b) l-C_{16}. RMSE in unit of $|\beta|$ as a function of the number of SE layers. Errorbars show the mean and standard deviation over ten times. Results for two noise environment (noisy CNOT gate and noisy single-qubit gate) and one ideal are compared. Fidelity is from previously published studies, [2] (red, CNOT and 1q), [3] (blue, CNOT), and [4](blue, 1q).

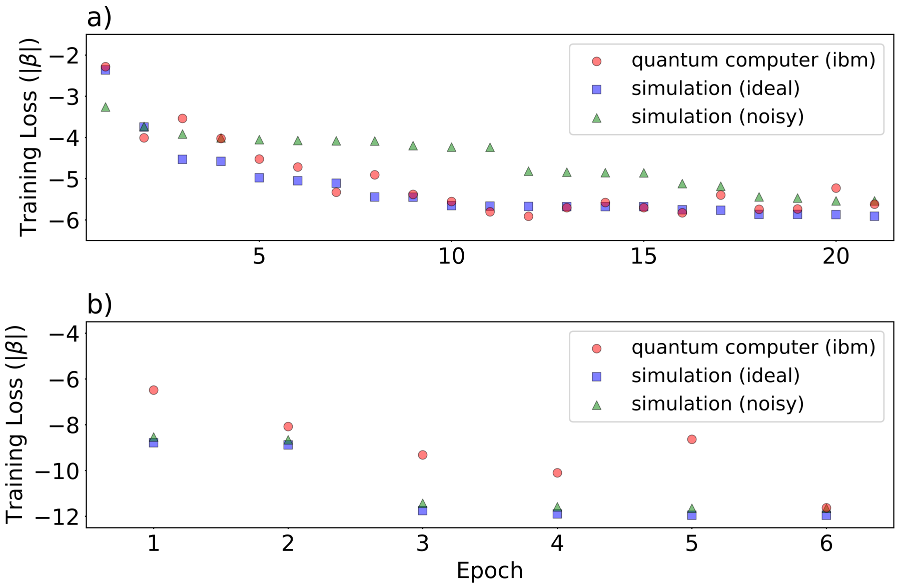

Figure S4: SSVQE training loss on an IBM quantum computer compared with ideal and noisy simulations for (a) c-C_4 and (b) c-C8 with one SE layer. The training loss is defined as the weighted sum of SSVQE energies, $\sum j = 0^{k-1} w_j E_j$. Experiments were performed on the IBM quantum computer, *ibm marrakesh* (January 31, 2026).[5] For each loss evaluation, 256 shots per circuit were used, resulting in a total of 64,512 shots for c-C_4 and 73,728 shots for c-C_8. For a fair comparison, the simultaneous perturbation stochastic approximation (SPSA) optimizer[6] was used for the QPU experiment (red), ideal simulation (blue), and noisy simulation (green). The parameters for the noisy simulations were chosen to reflect the reported median gate error rates of *ibm marrakesh*.

References

(1) Bergholm, V.; Izaac, J.; Schuld, M.; Gogolin, C.; Ahmed, S.; Ajith, V.; Alam, M. S.; Alonso-Linaje, G.; AkashNarayanan, B.; Asadi, A., et al. Pennylane: Automatic differentiation of hybrid quantum-classical computations. *arXiv preprint arXiv:1811.04968* **2018**.

(2) Dogan, E.; Rosenstock, D.; Le Guevel, L.; Xiong, H.; Mencia, R. A.; Somoroff, A.; Nesterov, K. N.; Vavilov, M. G.; Manucharyan, V. E.; Wang, C. Two-fluxonium cross-resonance gate. *Phys. Rev. Appl.* **2023**, *20*, 024011.

(3) Lin, W.-J.; Cho, H.; Chen, Y.; Vavilov, M. G.; Wang, C.; Manucharyan, V. E. 24 Days-Stable CNOT Gate on Fluxonium Qubits with Over 99.9% Fidelity. *PRX Quantum* **2025**, *6*, 010349.

(4) Marxer, F.; Mrożek, J.; Andersson, J.; Abdurakhimov, L.; Adam, J.; Bergholm, V.; Beriwal, R.; Chan, C. F.; Dahl, S.; Das, S. R., et al. Above 99.9% fidelity single-qubit gates, two-qubit gates, and readout in a single superconducting quantum device. *arXiv preprint arXiv:2508.16437* **2025**.

(5) IBM Quantum ibm_marrakesh, 2026.

(6) Spall, J. C. Multivariate stochastic approximation using a simultaneous perturbation gradient approximation. *IEEE Trans. Autom. Control.* **2002**, *37*, 332–341.
